# Comparison of Therapeutic Effect of Recombinant Tissue Plasminogen Activator by Treatment Time after Onset of Acute Ischemic Stroke

**DOI:** 10.1038/srep11743

**Published:** 2015-07-24

**Authors:** Hai-rong Wang, Miao Chen, Fei-long Wang, Li-hua Dai, Ai-hua Fei, Jia-fu Liu, Hao-jun Li, Sa Shen, Ming Liu, Shu-ming Pan

**Affiliations:** 1 Department of Emergency, Xinhua Hospital, Shanghai Jiao Tong University School of Medicine.

## Abstract

We aimed to compare the therapeutic effect of recombinant tissue plasminogen activator (rt-PA) administered at different time windows within the first 6 hours after onset of acute ischemic stroke (AIS). A retrospective analysis was performed of data collected from 194 patients who received rt-PA thrombolysis within 4.5 hours after AIS onset and from 29 patients who received rt-PA thrombolysis between 4.5–6 hours after AIS onset. The National Institutes of Health Stroke Scale (NIHSS) scores were statistically decreased in both groups (P < 0.05) at 24 hours and 7 days after onset. There was no statistical difference in the modified Rankin score or mortality at day 90 after treatment between the two groups (P > 0.05). In conclusion, AIS patients who received rt-PA treatment between 4.5–6 hours after onset were similar in therapeutic efficacy to those who received rt-PA within 4.5 hours after onset. Our results suggest that intravenous thrombolytic therapy for AIS within 4.5–6 hours after onset is effective and safe.

Cerebral stroke is the leading cause of disability and the second leading cause of death by disease in China^1^. About 6–7 million Chinese suffer from this disease with around 2 million new cases every year. Approximately 1.5 million patients will die each year from cerebral stroke. It dominates among diseases that cause disabilities; patients with acute ischemic stroke (AIS) account 60%–80% of all cerebral stroke patients.

Intravenous (IV) recombinant tissue plasminogen activator (rt-PA) is one of the most effective methods available for treatment of AIS. The National Institute of Neurological Disorders and Stroke (NINDS) was the first to report its efficacy in 1995, noting that 0.9 mg/kg rt-PA could significantly improve the clinical symptoms and outcome of patients with AIS within 3 hours of onset, an exceptionally early stage^2^. In 1996, IV rt-PA was approved by the FDA to treat AIS patients within 6 hours of the onset of symptoms. Unfortunately, although significant efforts have been made to increase the number of patients who arrive at treatment centers within 3 hours of the onset of symptoms, less than 1% of AIS patients in the United States receive rt-PA^3^.

It became evident that, in order to offer an opportunity for AIS patients who could not be treated within the preferred 3 hour time window to undergo thrombolytic therapy, extending the rt-PA treatment window was critical. The second trial of the European Cooperative Acute Stroke Study (ECASS II) tested the risk and benefit of such an extension: ECASS II was similar to ECASS I, except that the IV rt-PA dose was lowered to 0.9 mg/kg^4^,^5^. The results, released in 1998, showed that outcomes for AIS patients were improved when compared with ECASS I. Patients were stratified into 2 windows: 0–3 hours and 3–6 hours from symptom onset, with the majority of patients recruited in the 3–6 hour window. The primary favorable endpoint was a modified Rankin Scale (mRS) score of 0–1 at 3 months. Differences between treatment and placebo were similar in both time windows. The overall 3.7% absolute increase in improved outcomes in the IV rt-PA arm compared to the placebo arm was not statistically different (*P* = 0.277), but *post hoc* analysis using a modified favorable outcome definition of mRS score 0–2 produced an 8.3% absolute difference in favor of IV rt-PA (*P* = 0.024). The results of the Alteplase Thrombolysis for Acute Noninterventional Therapy in Ischemic Stroke (ATLANTIS) study, designed to test the use of rt-PA 3–5 hours after stroke onset, were published in 1999. ATLANTIS originally included patients treated with tPA from 0 to 6 hours from symptom onset, but was later modified to patients treated at 3 to 5 hours post onset (ATLANTIS B)^6^. The primary effective outcome measure was a 3-month United States National Institutes of Health Stroke Scale (NIHSS) score of 0 or 1 in ATLANTIS B. No significant benefit of rt-PA was found on the 3-month efficacy endpoints in patients treated between 3 and 5 hours.

The third ECASS trial (ECASS III) initially enrolled patients treated within 3 to 4 hours from symptom onset and later included those treated up to 4.5 hours^7^. Based on lessons learned from ECASS I, ECASS II, and ATLANTIS, criteria were deliberately selected to maximize the chances of favorable response. Patients with high stroke severity (NIHSS score >25), of extreme age (>80 years), with a combination of prior stroke and diabetes, and on any anticoagulant therapy regardless of international normalized ratio (INR) were excluded. The primary outcome measure, a 3-month mRS score 0 to 1, occurred in 52.4% of the IV rt-PA arm compared to 45.2% of the placebo arm (*P* = 0.04), a difference that remained statistically significant after adjustments for initial stroke severity and time from symptom onset to treatment. ECASS III was the first positive randomized phase III fibrinolysis trial for a treatment window extended beyond 3 hours. In 2008, the same year ECASS III was published, an observational study called Safe Implementation of Treatments in Stroke-International Stroke Thrombolysis Registry (SITS-ISTR) compared outcomes in patients treated between 3–4.5 hours or within 3 hours. There were no significant differences between the two cohorts for any outcome measure: rate of symptomatic intracerebral haemorrhage, or mortality and independence, indicating that rt-PA remained safe when given at 3–4.5 h after AIS onset^8^. Published in 2010, the Canadian Alteplase for Stroke Effectiveness Study (CASES) reported similar results: there were no differences between the 3–4.5 hour and <3 hour windows; both attained a 3 month mRS ≤1^9^. Liao XL *et al.* also reported that intravenous rt-PA treatment at 3 to 4.5 hours of symptom onset remains safe and effective in Chinese patients with AIS in 2013^1^. Finally, the third International Stroke Trial (IST-3) studied the efficacy of a 0–6 hour fibrinolysis window, and confirmed the benefit of IV rt-PA. An Oxford Handicap Score (OHS) of 0 or 1 was considered to be a favorable outcome, and was achieved by 24% of the rt-PA group and 21% of the control group (P = 0.018)^10^. IST-3 did not report on outcomes of treatment between 4.5–6.0 hours from onset of AIS symptoms.

Since 2008, about 300 AIS patients have received rt-PA treatment in our department. However, some patients were not sent to hospital immediately after symptoms occurred, for various reasons including lack of understanding of the disease. For those patients who presented within 4.5–6 hours after onset of AIS symptoms, a selective emergency thrombolysis treatment was administered after thorough evaluation of the patient’s condition, relevant risks, and requirements of patients and their families. In this article, we summarize the results of rt-PA thrombolysis treatment in our hospital of AIS patients within 4.5 hours and between 4.5–6 hours after onset of symptoms.

## Materials and Methods

### Inclusion Criteria

The study protocol was approved by the ethics committees of Xinhua Hospital, Shanghai Jiao Tong University School of Medicine. The study was carried out in accordance with the Helsinki Declaration. AIS was diagnosed in accordance with the standards for diagnosis of cerebral infarction from the Fourth National Academic Meeting of Cerebrovascular Disease in 1995, and in accordance with the National Guideline for Diagnosis and Treatment of Acute Ischemic Stroke 2010 of China, except the time frame of treatment was extended from 4.5 to 6 hours after onset of AIS symptoms^11^. Inclusion criteria for subjects were: (1) age: 18–80 years; (2) physical signs of brain function damage persisted for 1 hour, paralysis < degree 3; (3) intracranial hemorrhage excluded by cerebral CT, and no massive cerebral infarction indicated by imaging changes.

### Exclusion Criteria

We followed the National Guideline for Diagnosis and Treatment of Acute Ischemic Stroke 2010 of China^12^ to exclude: (1) patients with intracranial hemorrhage including suspicious hemorrhage in subarachnoid space, head trauma in the previous 3 months, gastrointestinal hemorrhage or urinary system hemorrhage in the previous 3 weeks, major surgery in the previous 2 weeks, arterial puncture sites where hemostasis by compression was not convenient to perform in the previous week; (2) patients with cerebral infarction or myocardial infarction in the previous 3 months, excluding old lacunula infarction without sequela of neurologic functional symptoms; (3) patients with severe functional insufficiency of cardiac, hepatic or renal systems or with diabetes mellitus; (4) signs of active hemorrhage or trauma (such as fracture) observed upon physical examination; (5) patients who took oral anti-coagulation drugs, and INR >15; patients who received heparin therapy within 48 hours (APrT exceeding normal range); (6) platelet count below 100 × 10^9^/L, blood glucose <2.7 mmol/L; (7) systolic blood pressure >180 mmHg or diastolic pressure >100 mmHg; (8) pregnant or lactating female patients; (9) non-cooperative patients.

### General Information

Two hundred twenty-three AIS patients received rt-PA thromoblysis therapy at our institution between June 2010 and October 2014. One hundred ninety-four patients received thromoblysis therapy within 4.5 hours of symptom onset, and 29 patients received the same treatment within 4.5 to 6 hours of the onset of AIS symptoms.

### Treatment Method

Rt-PA 0.9 mg/kg (maximum dose 50 mg) was administered. Initially, 10% (1 minute) was intravenously injected, followed by the remaining 90% continuously infused by an intravenous pump over 60 minutes. Thrombolysis treatment was discontinued if severe headache, acute elevation of blood pressure, nausea or vomiting ensued. Head CT scans were re-examined to detect any cerebral hemorrhage. No anti-coagulation drugs or aspirin were administered within 24 hours after thrombolysis treatment. Platelet and blood coagulation function were re-examined immediately if cerebral hemorrhage appeared, and fresh plasma or 1 U platelets were then transfused. Four parameters of blood coagulation and head CT were examined 24 hours after thrombolysis treatment. Low molecular weight heparin was injected subcutaneously for 7 days, and administered orally thereafter. Treatments to improve brain cell metabolism were also administered at the same time.

### Efficacy Evaluation

The NIHSS was used to quantify impairment before treatment and 24 hours and 7 days after thrombolysis treatment. The mRS was used to evaluate patients’ daily functional abilities at 90 days after thrombolysis treatment.

### Statistical Method

Data was expressed as mean ± standard deviation (SD), median, and interquartile range (IQR). Continuous variables were analyzed by Student’s *t* test. Discrete variables were compared by Chi Square tests, Signed rank sum test and Mann-Whitney U test. A *P* value <0.05 was considered as statistically significant. Statistical analysis of the data was done using the Statistical Package for the Social Sciences (SPSS) version 17 or SAS6.12.

## Results

### General Information

The mean age of patients who accepted treatment <4.5 hours after onset was 64.4 ± 10.0 years, and the mean age of patients who accepted treatment between 4.5–6 hours after onset was 66.8 ± 9.2 years. There was no statistical difference between the two groups in age, gender, or AIS risk factors such as ratio of patients with hypertension, diabetes mellitus or atrial fibrillation (P > 0.05; Table 1). The patients in the two groups were therefore comparable.

### Comparison of Therapeutic Effect in Treatment Windows

**Figure 1:**
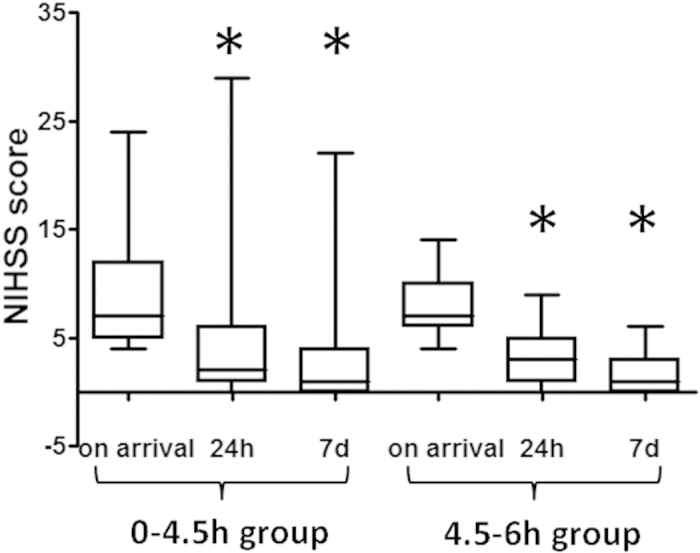
Comparison between NIHSS scores of the two groups before and after thrombolysis: *indicates P < 0.05.

The patient mortality rate was 5.2% (10 of 194) in the 0–4.5 hour group, and 6.9% (2 of 29) in the 4.5–6 hour group, with no statistically significant difference (i^2^ = 0.1503, P = 0.6982). All deaths were caused by cerebral hemorrhage except one patient in the 0–4.5 hour group who died of upper gastrointestinal hemorrhage and another patient in the 0–4.5 hour group who died of cardiac arrest. Death data were therefore excluded from statistical analysis of the therapeutic effect. The median baseline NIHSS score in the 0–4.5 hour group was 7.0, IQR was [5.0–12.0], which decreased to 2.0 [1.0–6.0] at 24 hours after thrombolysis treatment, a statistically significant reduction (Z = −10.3306, P < 0.0001). The NIHSS score of this group decreased further to 1.0 [0–4.0] at 7 days after thrombolysis treatment, also a statistically significant reduction compared with the baseline score (Z = −12.4174, P <0.0001; Fig. 1). For patients in the 4.5–6 hour group, the baseline NIHSS score was 7.0 [6.0–10.0], which decreased to 3.0 [1.0–5.0] at 24 hours after thrombolysis treatment (Z = −4.8022, P < 0.0001), and decreased to 1.0 [0–3.0] at 7 days after treatment (Z = −6.0832, p <0.0001 compared to the baseline score; Fig. 1). These results indicated that patients in both groups benefited from rt-PA treatment.

**Figure 2:**
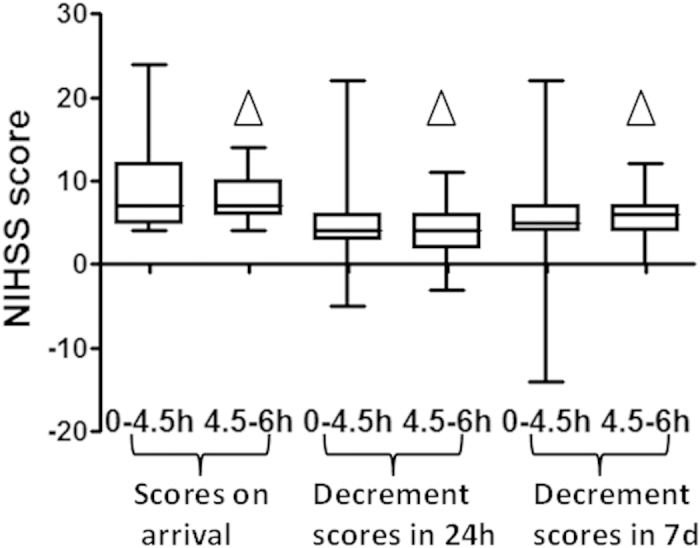
Comparison of improvement of NIHSS scores of patients after thrombolysis in the two groups: ^△^ P > 0.05.

There was no statistical difference in baseline NIHSS scores between patients in the 4.5–6 hour group and in the 0–4.5 hour group (Z = −0.3418, p = 0.7325). Moreover, there was no statistical difference in the improvement of NIHSS scores at 24 hours and 7 days after thrombolysis treatment, indicating that the effect of thrombolysis on patients in the two groups was very similar. The NIHSS score improved 4 [3.0–6.0] in the 0–4.5 hour group at 24 hours after thrombolysis, and 4.0 [2.0–6.0] (Z = −0.5770, P = 0.5639) in the 4.5–6 hour group. The NIHSS scores improved 5.0 [4.0–7.0] in the 0–4.5 hour group at 7 days after thrombolysis, while for the NIHSS scores in the 4.5–6 hour group improved 6.0 [4.0–7.0] (Z = 0.5165, P = 0.6055; Fig. 2).

**Figure 3:**
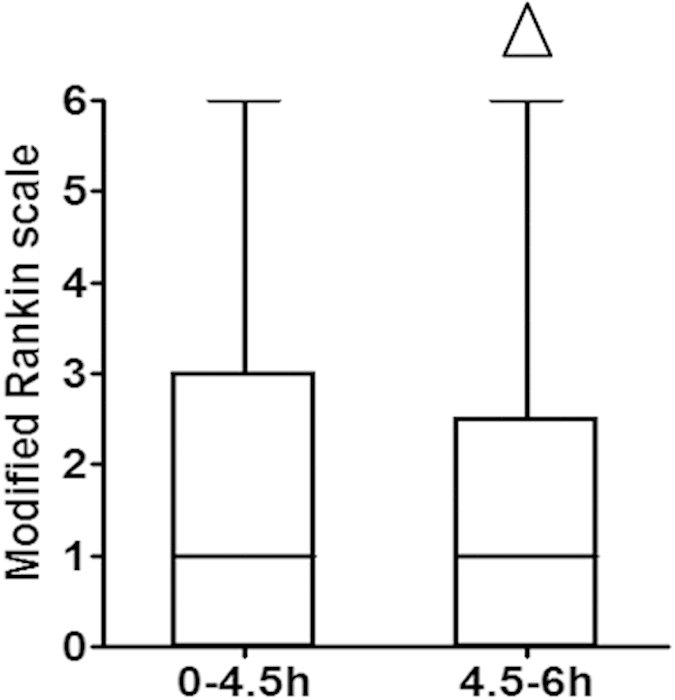
Modified Rankin Scale scores of patients in the two groups 90 days after thrombolysis: ^△^ indicates P > 0.05.

We used the mRS to assess the daily functional abilities of patients at 3 months after thrombolysis treatment. The 3-month mRS for patients in the 0–4.5 hour group was 1.0 [0–3.0], and 1.0 [0–2.5] for patients in the 4.5–6 hour group (Z = 0.2770, P = 0.7818), an insignificant difference (Fig. 3). The overall improved outcomes (3-month mRS score 0 to 1) in the 0–4.5 hour group was 53.1% (103 of 194), compared to 51.7% (15 of 29) in the 4.5–6 hour group (χ^2^ = 0.0190, P = 0.8905), indicating that the comprehensive daily functional abilities and independence of patients in the two groups were similar.

### Adverse Reactions and Complications

The incidence of symptomatic intracerebral hemorrhage was 10.8% (21 of 194) and mortality was 5.2% (10 of 194) in the 0–4.5 hour group. In this group, 8 patients died because of symptomatic intracerebral hemorrhage, one suffered from fatal upper gastrointestinal hemorrhage and another patient died of cardiac arrest. In the 4.5–6 hour group, 5 patients developed symptomatic intracerebral hemorrhage (5 of 29, for an incidence rate of 17.2%), and 2 of whom were died (6.9% of total). There was no statistical difference in incidences of either cerebral hemorrhage or mortality (χ^2^ = 1.0085, p = 0.3153 and χ^2^ = 0.4529, p = 0.5010, respectively).

## Discussion

AIS results from the sudden interruption of arterial blood flow to a dependent area of the brain parenchyma. A central zone of ischemic tissue known as *the core* is completely deprived of critical oxygen and glucose delivery, and irreversible neuronal injury begins within minutes. Surrounding the core are areas of markedly decreased perfusion where “stunned” brain parenchyma receives a diminished blood supply from cerebral collateral vessels and potentially from residual arterial flow. This area, known as the *ischemic penumbra*, contains many viable nerve cells and is potentially salvageable if significant arterial flow can be quickly restored; thus, it is the therapeutic target of reperfusion stroke therapies^12^,^13^.

Emergency rt-PA thrombolysis treatment has become one of the most effective therapeutic options available since it was first reported in 1995. It can significantly improve the clinical outcomes of patients with AIS if applied within 3 hours^2^. Extending the therapeutic window for thrombolysis is an important strategy in maximizing the proportion of patients treated. The ECASS III study in 2008 showed that rt-PA administered within 3 to 4.5 h after symptom onset significantly improved clinical outcomes in patients with AIS^7^. It is widely acknowledged at present that rt-PA thrombolysis treatment administered within 4.5 hours after occurrence of symptoms is safe and effective^1^,^7^,^9^,^14^,^15^. In 2012, the IST-3 study provided evidence that the therapeutic window for thrombolysis may extend to 6 hours after AIS commences^10^. Research indicates that the penumbra area is detectable in about 70% of patients who accept examination within 6 hours after stroke, which means that it would be possible to expand the window of thrombolysis treatment up to 6 hours^16^. Based on the factors above, we performed rt-PA thrombolysis on patients who presented 4.5–6 hours after AIS onset.

In this study, the baseline NIHSS scores in the 4.5–6 hour group and 0–4.5 hour group were no statistical difference, and the NIHSS scores of patients in both groups improved significantly after thrombolysis treatments. After rt-PA treatment, there was no statistical difference between the two groups in the improvement of NIHSS score at 24 hours and 7 days. The 3-month mRS score of 0 to 1 in the 0–4.5 hour group was 53.1% compared to 51.7% in the 4.5–6 hour group, an insignificant difference, indicating that the patients’ comprehensive daily functionality is similar in the two groups.

Although the symptomatic intracerebral hemorrhage rate was 17.2% in the 4.5–6-hour group compared to 10.8% in the 0–4.5-hour group, there was no statistically significant difference between them. The symptomatic intracerebral hemorrhage rate in the 0–4.5 hour group (10.8%) was similar to that of the thrombolysis group of NINDS (6.4%), ECASS III (7.9%) and IST-3 (7%), while the symptomatic intracerebral hemorrhage rate in the 4.5–6 hour group (17.2%) was higher than that of the thrombolysis group of these trials^2^,^7^,^10^. The 90-day mortality rate was 5.2% in the 0–4.5 hour groups and 6.9% in the 4.5–6 hour group, which were similar to that of the thrombolysis group of ECASS III (6.7%), and lower than NINDS (7.7%) and IST-3 (27%)^2^,^7^,^10^. Therefore, we consider that it is safe to perform rt-PA thrombolysis within 4.5–6.0 hours after AIS occurs.

Our team had thrombolysis experience in more than 300 AIS cases since 2008, and we had been awarded the ‘Thrombolysis Pioneer Award’ by the Stroke Quality Control Center of the Ministry of Health of China in 2013. So our results should be interpreted with caution as it they might be applicable to other stroke centers; however, the mantra still holds true: the earlier thrombolysis treatment begins, the better the effect is likely to be and the less risk there is^17^. We recommend that in those hospitals with experience in rt-PA thrombolysis and after a patient’s condition has been thoroughly evaluated, the thrombolysis time window can be extended up to 6 hours after onset of symptoms for AIS patients who have low NIHSS scores.

## Additional Information

**How to cite this article**: Wang, H. *et al.* Comparison of Therapeutic Effect of Recombinant Tissue Plasminogen Activator by Treatment Time after Onset of Acute Ischemic Stroke. *Sci. Rep.* **5**, 11743; doi: 10.1038/srep11743 (2015).

## Figures and Tables

**Table 1:** Demographic and baseline characteristics of patients in the two treatment windows.

	Age (years)	Gender (male/female)	Hypertension(%)	Diabetes (%)	Atrial Fibrillation (%)
<4.5 h	64.4 ± 10.0	118/76	73.7(143/194)	24.7(48/194)	21.1(41/194)
4.5−6 h	66.8 ± 9.2	22/7	79.3(23/29)	17.2(5/29)	34.5(10/29)
**Statistical**	t = −1.24	χ2 = 2.4414	χ2 = 0.4157	χ2 = 0.7834	χ2 = 2.5485
P value	0.2156	0.1182	0.5190	0.3761	0.1104
